# STING expression in pituitary neuroendocrine tumour immune microenvironments

**DOI:** 10.1530/ERC-26-0385

**Published:** 2026-09-28

**Authors:** Paul Benjamin Loughrey, Aoife McArdle, Amélie Viratham Pulsawatdi, Kris D McCombe, Christine Greene, Hannah James, Estelle G Healy, Fionn Corr, Ella Ross, Stephen Cooke, Steven J Hunter, Márta Korbonits, Stephanie G Craig, Jacqueline A James

**Affiliations:** ^1^ Johnston Cancer Research Centre, Queen’s University Belfast, Queen’s University Belfast, Belfast, United Kingdom; ^2^ Department of Endocrinology, Beaumont Hospital, Dublin, Ireland; ^3^ Northern Ireland Biobank, Future Medicines Institute, Queen’s University Belfast, Belfast, United Kingdom; ^4^ Department of Cellular Pathology, Belfast Health and Social Care Trust, Belfast, United Kingdom; ^5^ Department of Neurosurgery, Belfast Health and Social Care Trust, Belfast, United Kingdom; ^6^ Regional Centre for Endocrinology and Diabetes, Belfast Health and Social Care Trust, Belfast, United Kingdom; ^7^ Department of Endocrinology, William Harvey Research Institute, Barts and the London School of Medicine, Queen Mary University of London, London, United Kingdom; ^8^ Regional Molecular Diagnostic Service, Belfast Health and Social Care Trust, Belfast, United Kingdom

**Keywords:** pituitary, digital pathology, multiplex immunofluorescence, tumour immune microenvironment, STING, AIP

## Abstract

The pituitary neuroendocrine tumour (PitNET) immune microenvironment (IME) has become a recent focus of research interest. Multiplex immunofluorescence combined with digital image analysis is a powerful technique to interrogate the IME. Processes such as DNA damage response and cellular senescence have been implicated in somatotroph tumour pathogenesis, and these have in turn been linked with immunological responses to neoplasia. The objectives of this study were to compare the immune cell infiltrate in PitNETs to normal pituitary, investigate how the PitNET-IME may vary according to treatment with somatostatin receptor ligand, tumour invasiveness, tumour proliferation, and *AIP* mutation status. Two bespoke multiplex panels were developed and applied to a range of PitNET subtypes before being interrogated with deep learning algorithms and QuPath image analysis software. Tumours had significantly higher total macrophage infiltrate (*P* < 0.001), STING+ macrophages (*P* < 0.001) and STING positivity (*P* < 0.001) compared with normal pituitary tissue. Macrophage infiltration and STING positivity were positively correlated with tumour Ki-67 proliferation index (*r*_s_ = 0.305, *P* = 0.003; and *r*_s_ = 0.263, *P* = 0.010, respectively). Results suggested that *AIP*-mutated PitNETs may have higher CD8+ T-lymphocyte infiltrate, macrophage infiltrate, including STING+ macrophages and overall STING positivity compared with *AIP*-negative tumours. The application of machine learning to multiplex immunofluorescence images identifies macrophages and STING as upregulated in these subgroups of functioning PitNETs. This immunological response may indicate that DNA damage, cellular senescence and autophagy could be features of these tumours.

## Introduction

Pituitary neuroendocrine tumours (PitNETs) are a heterogeneous group of neoplasms arising from the anterior pituitary gland. Functioning tumours can secrete growth hormone (GH), prolactin and/or thyroid-stimulating hormone (TSH) and result in clinical syndromes including acromegaly, hyperprolactinaemia or hyperthyroxinaemia. Despite their typically indolent behaviour, these tumours result in significant comorbidity and mortality *via* local neurovascular compromise, hypopituitarism and hormone excess. A few millimetres of growth can result in visual impairment or incurable disease despite their indolent behaviour. At the other end of the clinical spectrum are aggressive PitNETs, which are associated with rapid growth, local invasion and potentially metastatic disease (1). Treatment options for aggressive PitNETs are limited, but immunotherapies have resulted in promising therapeutic response in some cases (2). Consequently, recent research has focused on the PitNET immune microenvironment (IME) in an effort to identify novel biomarkers and therapeutic targets (3).

The tumour immune microenvironment is a complex ecosystem of tumour cells, immune cells, extracellular matrix and vasculature (4). Each component of the immune microenvironment can be anti-tumourigenic or pro-tumourigenic (5). Furthermore, immune system responses can be considered as innate or adaptive. Immune cells involved in the immune response to neoplasia include T lymphocytes, B lymphocytes and macrophages. Early examination of the PitNET-IME utilising brightfield microscopy and manual quantification did not find that immune cell infiltrate was particularly notable in PitNETs (6). Contemporary studies using similar techniques have made more interesting insights, correlating PitNET-IME immune cell infiltrate with clinical features such as tumour size, tumour invasion, functional status, treatment response, angiogenesis and *AIP* mutation status (3, 7, 8, 9, 10, 11).

Alongside PitNET-IME immune cell infiltrate, there has been heightened interest in immune-regulatory pathways in PitNETs, particularly programmed death-ligand 1 (PD-L1)/programmed cell death protein 1 (PD-1) and cytotoxic T-lymphocyte-associated protein 4 (CTLA-4) immune checkpoints. One immune signalling pathway which is particularly novel in human PitNETs is the innate immune cyclic guanosine monophosphate–adenosine monophosphate (cGAMP) synthase (cGAS) stimulator of interferon genes (STING) pathway. The cGAS–STING pathway is an intracellular DNA-sensing pathway activated by foreign DNA or damaged self-DNA. STING is a transmembrane protein that is located on the endoplasmic reticulum. Upon activation by cGAMP, STING undergoes a conformational change to activate interferon regulatory factor 3 and nuclear factor ΚB, thus inducing the expression of interferon genes and pro-inflammatory chemokines. In addition, once activated, STING translocates to perinuclear microsomal compartments (12). The cGAS–STING pathway has been described as essential for DNA damage-induced cellular senescence, a process which has been shown to be a feature of somatotroph tumours (13, 14, 15). The cGAS–STING pathway also has intimate links with the process of autophagy, and the inhibition of autophagy has been found to suppress cell proliferation and GH production in rat GH4 cells (16, 17). Recent data indicate that STING is predominantly expressed on macrophages within the PitNET-IME (18). Finally, previous data have indicated that chemokine ligand 5 is an important cytokine in the pathogenesis of *AIP*-mutated PitNETs and is associated with macrophage infiltration of these tumours (7). The cGAS–STING pathway is a key regulator of chemokine ligand 5 in DNA damage response-deficient breast cancer (19). Given the existing links with cGAS–STING, DNA damage response, cellular senescence and autophagy, it was hypothesised that the innate immune cGAS–STING signalling pathway may have roles in PitNET pathogenesis.

The aim of this study is to utilise multiplex immunofluorescence, digital image analysis and artificial intelligence techniques to perform simultaneous PitNET-IME biomarker assessment in a population-representative cohort of thyrotroph tumours, lactotroph tumours and PitNETs associated with GH excess.

## Materials and methods

### Clinicopathological data

Clinicopathological data were retrieved during the study period from 1 January 2000 to 19 July 2019 and can be considered a population-representative cohort. The data were retrospectively retrieved from patient records and held within an audit and quality improvement database (approval 6057). The linkage of de-identified clinicopathological data and samples was undertaken using established procedures between the Northern Ireland Biobank and Northern Ireland Cancer Registry.

Acromegaly was diagnosed according to internationally accepted clinical guidelines; preoperative diagnostic investigations included serum insulin-like growth factor 1 levels, random GH levels and oral glucose tolerance testing GH levels (20, 21). Biochemical confirmation of GH excess was followed by pituitary radiological imaging. Lactotroph tumours were diagnosed according to internationally recognised clinical guidelines with measurement of serum prolactin levels and pituitary radiological imaging (22). Thyrotroph tumours were diagnosed in accordance with internationally recognised clinical guidelines with measurement of thyroid-stimulating hormone, free T4, free T3, serum glycoprotein hormone alpha subunit, T3 suppression testing, sex hormone-binding globulin measurement, thyroid autoantibodies and pituitary radiological imaging (23). Following diagnosis, PitNETs in this study cohort were resected in a single centre. Resection was undertaken by one of two pituitary neurosurgeons, and the subsequent histopathological diagnosis was made by one of two pituitary neuropathologists. Histopathological immunohistochemistry (IHC) included haematoxylin and eosin, Ki-67 and anterior pituitary hormones (GH, prolactin, TSH, follicle-stimulating hormone, luteinising hormone, adrenocorticotrophic hormone). Postoperative histopathological reports were reviewed, and anterior pituitary hormone expression data were extracted. Due to the retrospective nature of the cohort, pituitary transcription factor expression was not available. Existing histological reports were reviewed, and if tumours were necrotic, they were omitted from the cohort.

Microtumours were defined as maximal tumour diameter <10 mm, and macrotumours were defined as ≥10 mm on preoperative magnetic resonance imaging. Invasive tumours were those which had invaded the cavernous sinus or sphenoid sinus based on magnetic resonance imaging and/or intraoperative assessment. Data on whether patients had received preoperative long-acting release preparations of somatostatin receptor ligand were retrieved and categorised as a binary yes or no variable. Where available, *AIP* mutation status was extracted from the clinicopathological database and expressed as a binary yes or no variable.

### Patient samples

Use of anonymised surplus formalin-fixed paraffin-embedded samples for the multiplex immunofluorescence analysis was granted in accordance with standard operating procedures of the Northern Ireland Biobank. Samples of pituitary tissue adjacent to resected Rathke’s cleft cysts were used as a normal control.

### Panel development and optimisation and preparation of pituitary samples for multiplex immunofluorescence

Following literature review, biomarkers of the immune microenvironment were selected. Antibodies and concentrations were as follows: CD3; 2GV6 (Ventana, USA, catalogue no. 790-4341) Epitope retrieval (ER) 1 30 min 1:6, CD4; SP35 (Ventana, 790-4423) ER1 30 min 1:4, CD8; C8/144B (Dako M7103) ER1 30 min 1:400, CD20; L26 (Dako, Denmark, MO755) ER1 30 min 1:400, Ki-67; MIB1 (Dako M7240) ER2 20 min 1:75, CD68; 514H12 (Novocastra, UK, NCL-L-CD68) ER1 30 min 1:4, STING; Poly (ProteinTech, USA, 19851-1-AP) ER1 30 min 1:2,000, PD-L1; SP263 (Ventana 790-4905) ER2 20 min 1:2, CTLA-4; BSB-88 (BioSB, USA, BSB2885) ER2 10 min 1:50, synaptophysin; 27G12 (Novocastra, NCL-L-SYNAP-299) ER1 30 min 1:200.

Sections from formalin-fixed paraffin-embedded samples were cut at 3 µm thickness. Optimal antibody concentrations, ER times, fluorophore-antibody pairing and antibody order for optimisation of MPs were determined as previously described (24). These were confirmed using dropout controls. Both tyramide signal amplification and DAB-mediated IHC are reliant on peroxidase and so the staining order from the conventional chromogenic IHC was directly transferred to the multiplex immunofluorescence (MIF) panel application. MOTiF Opal-antibody pairings were guided by existing IME literature, with those biomarkers expected to co-localise paired with spectrally different Opal dyes (Fig. 1A and B). More intense MOTiF Opal dyes were paired with biomarkers which were expected to be expressed at a low frequency (Fig. 1A and B).

**Figure 1:**
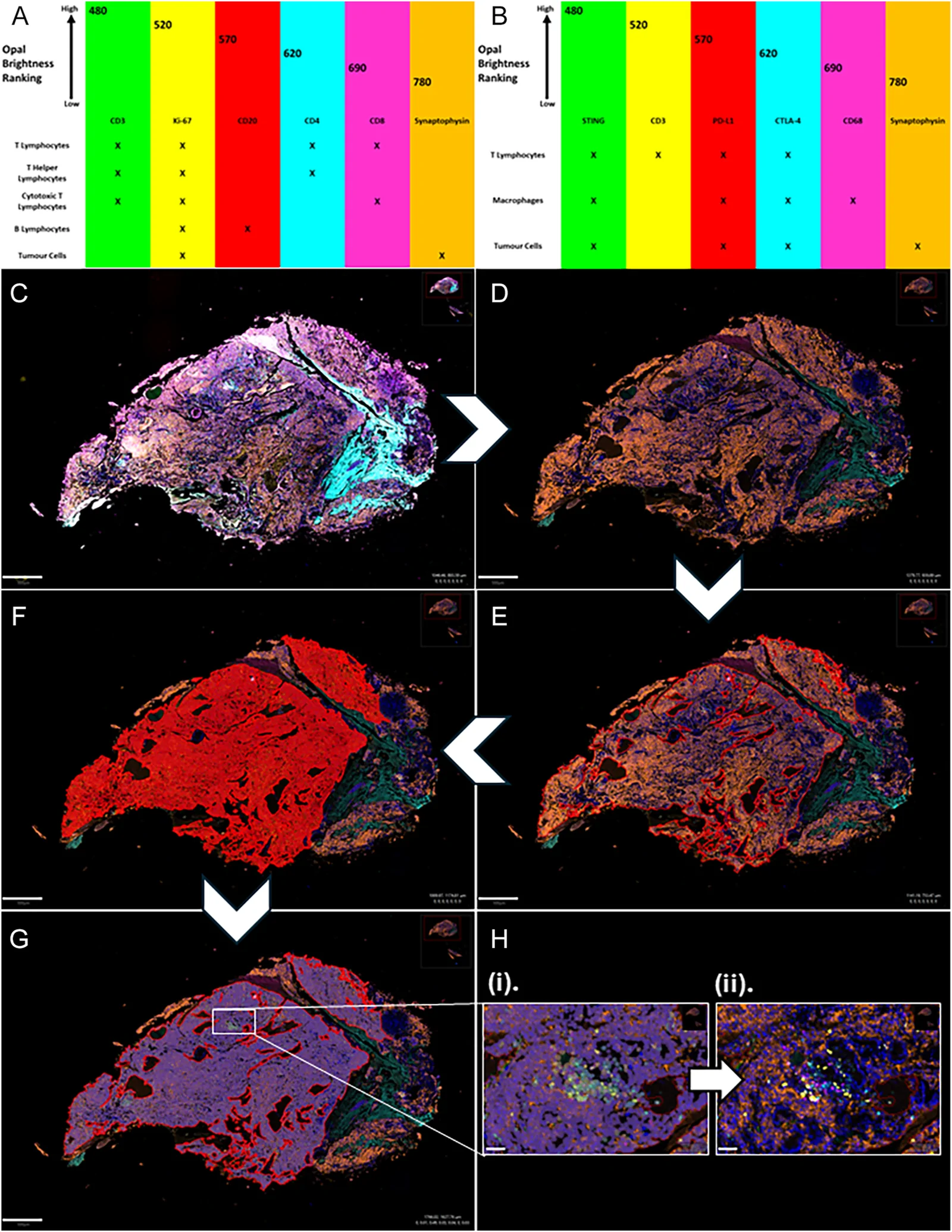
(A) Opal dye pairing for MP1. (B) Opal dye pairing for MP2. (C) Stitched unmixed image of somatotroph tumour stained for MP1 prior to optimisation of brightness and contrast. (D) The same image as in (A) with brightness and contrast adjusted to optimise visualisation of positively staining cells. (E) Annotation of tumour tissue using QuPath (red lines). (F) Somatotroph tumour following the application of the StarDist deep learning algorithm for 2D and 3D nucleus detection (26). (G) Somatotroph tumour with composite classifier applied. (H) Focus of immune cell infiltrate in Fig 1E enlarged with QuPath mask on (i) and QuPath mask off (ii). CD3+ cells, lime; CD4+ cells, cyan; CD8+ cells, magenta; CD20+ cells, red; Ki-67+ cells, yellow; synaptophysin+ cells, orange. Images (C, D, E, F, G) at ×2 magnification, scale bars 500 μm. Image (H) at ×20 magnification, scale bars 50 μm.

### Digital image acquisition

Multiplex immunofluorescence sections were scanned using tile scanning on the PhenoImager HT™ at ×20 magnification and spectrally unmixed in InForm™ using synthetic libraries provided within the software (25). After spectral unmixing, a composite whole slide MIF image was created from spectrally unmixed tiles on QuPath using custom scripting to form Tag Image File Format (TIFF) images of whole-face sections amenable to digital image analysis.

### Image analysis

TIFF images were examined using QuPath version 0.2.3 (26). Brightness and contrast were adjusted to optimise visualisation of positively staining cells (Fig. 1B and C). Areas of tumour were manually annotated before the StarDist deep learning algorithm for 2D and 3D nucleus detection was applied (Fig. 1E and F) (27). Single-measurement cell classifiers were created using built-in QuPath machine learning functions, with up to three positivity thresholds set for each biomarker (low, medium, high). This was a random forest classifier that uses both the pixel intensity across markers expressed by each cell and shape features of the nucleus to mitigate excessive autofluorescence in samples. Composite classifiers were created by combination of single measurement classifiers, and optimal composite classifiers were applied to each image (Fig. 1G and H). Data were extracted from QuPath to a Microsoft Excel file in advance of statistical analysis. For the purpose of this analysis, the data from CD3, CD4, CD8, CD20, CD68, Ki-67, STING and synaptophysin are presented.

### Statistical analysis

Statistical analysis was undertaken with *GraphPad Prism 10.2.3*. Positively staining cells were expressed as a proportion of the total cells detected, apart from the Ki-67 proliferation index (PI). Ki-67 PI was expressed as the fraction of Ki-67-positive tumour cells divided by the total number of tumour cells detected, multiplied by 100 to give a percentage. Due to the small proportions being analysed and the presence of zero values, the data were transformed to square root (28). Normality of data was assessed using the Shapiro–Wilk test. Correlation was assessed using Spearman’s rank correlation. The Wilcoxon matched-pairs signed-rank test was used to compare biomarker expression between matched samples. The Mann–Whitney test was used to compare biomarker expression between two unmatched groups and the Kruskal–Wallis test was used to compare biomarker expression between three or more unmatched groups. A *P* value < 0.05 was considered significant.

## Results

### Demographics

The demographics of this retrospective cohort are summarised in Table 1. A total of 99 resection samples were successfully retrieved for analysis with multiplex panel (MP) 1 and 102 for MP2 (Fig. 2A and B).

**Table 1:** Demographics of patients with tumours analysed for biomarkers MP1 and MP2.

	MP1 (*n* = 99)			MP2 (*n* = 102)		
	GH Excess *n* = 83	Prl *n* = 10	Thy *n* = 6	GH Excess *n* = 87	Prl *n* = 10	Thy *n* = 5
Age at diagnosis (y (range))	48 (19–77)	38 (18–71)	46 (27–65)	48 (19–77)	38 (18–71)	45 (27–48)
Unknown (*n*)	2	0	0	3	0	0
Sex (*n*)						
Female	28	6	5	30	6	4
Male	55	4	1	57	4	1
Tumour size (*n*)						
Macro	62	5	5	64	6	4
Micro	16	4	1	17	4	1
Unknown	5	1	0	9	1	0
Extension/invasion (*n*)						
Suprasellar	35	6	4	38	6	4
Sphenoid	17	2	2	18	2	2
Cavernous	24	4	1	24	4	1
Unknown	7	0	0	9	0	0
Hormone IHC (*n*)						
GH	51	0	0	53	0	0
PRL	0	8	0	0	8	0
TSH	0	0	1	0	0	1
GH + PRL	19	1	0	19	1	0
GH + TSH	5	0	5	5	0	4
Hormone negative	2	1	0	4	1	0
ACTH	1	0	0	1	0	0
Unknown	5	0	0	5	0	0

Age at diagnosis is displayed as median and range. GH, growth hormone; Prl, lactotroph tumour; Thy, thyrotroph tumour; IHC, immunohistochemistry; ACTH, adrenocorticotrophic hormone.

**Figure 2:**
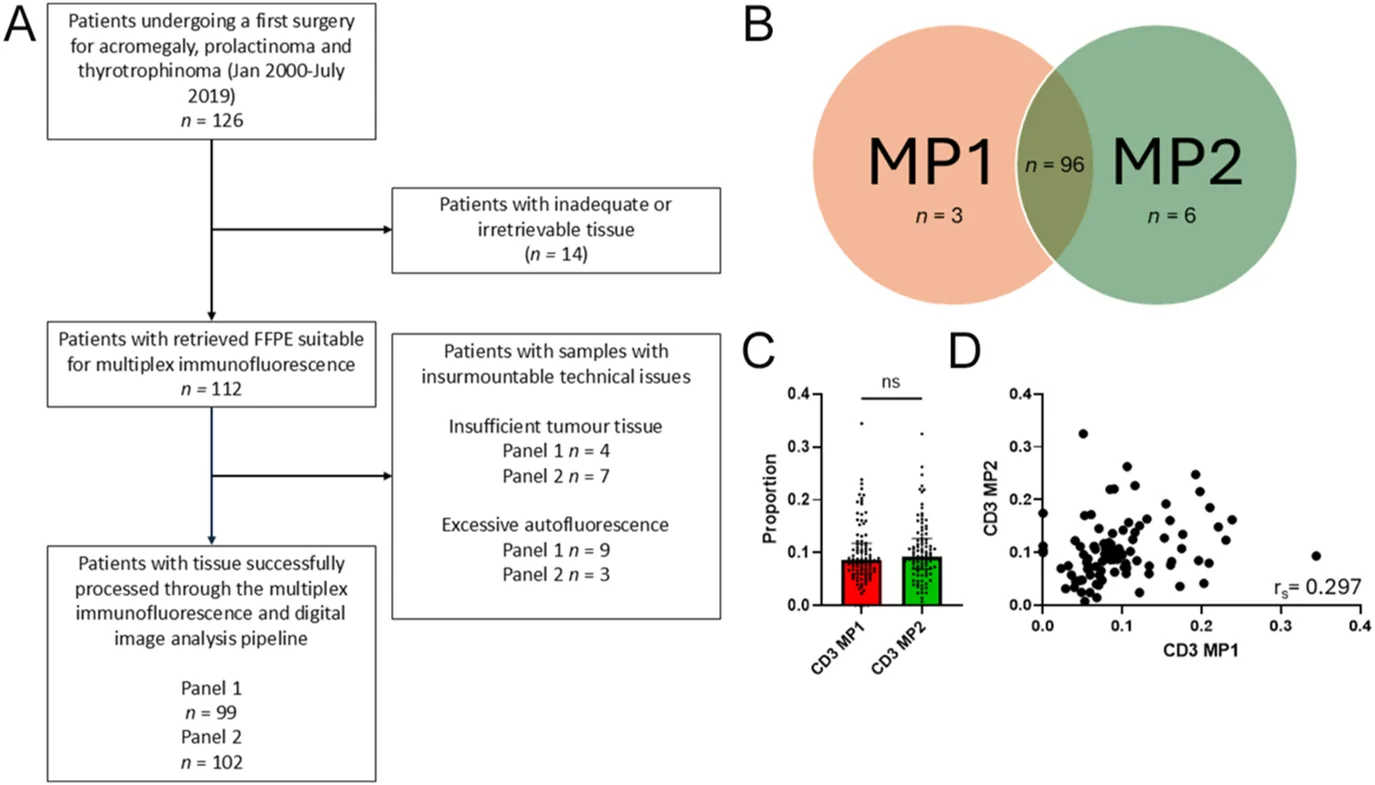
(A) STROBE diagram detailing the selection process in generating the cohort of samples used in this study. (B) Venn diagram showing the number of samples interrogated for MP1 and MP2. (C) Scatter plot comparing proportions of CD3 cells detected in MP1 to MP2 (*n* = 95), *P* = 0.288. Error bars indicate IQR. (D) Correlation plot showing a weak positive correlation between cell proportions of CD3 cells detected in MP1 with MP2 (*n* = 95), *r*_s_ = 0.297, *P* = 0.003. FFPE, formalin-fixed paraffin-embedded tissue.

### Comparison of CD3 assessment between panels

Both MPs contained the T-lymphocyte marker CD3. There was no significant difference in CD3 expression between matched tumour samples (Fig. 2C). There was a weak positive correlation in CD3 expression in panel 1 and panel 2 (*r*_s_ = 0.297, *P* = 0.003, Fig. 2D).

### IME immune cell infiltrate in tumours compared with normal pituitary

When considering MP1 biomarkers, there were no significant differences in total T and B lymphocyte infiltrate (CD3+, CD4+, CD8+ and CD20+ combined) compared with normal pituitary (*P* = 0.687, Supplementary Fig. 1A (see section on Supplementary materials given at the end of the article)). Similarly, there were no significant differences when considering CD3+ (*P* = 0.723), CD4+ (*P* = 0.876), CD8+ (*P* = 0.697) T lymphocyte and CD20+ B lymphocyte (*P* = 0.176) infiltration in tumours compared with normal pituitary (Supplementary Fig. 1B, C, D, E).

Although there were not significantly higher levels of CD3+ T lymphocytes in tumours compared with normal pituitary (*P* = 0.305), when considering CD3+ STING+ T lymphocytes, there were significantly more of this T lymphocyte subset in tumours compared with normal pituitary (*P* < 0.001) (Fig. 3A, B, C). There were also significantly more CD68+ (*P* < 0.001), CD68+ STING+ (*P* < 0.001) macrophages and higher STING expression in tumour cells (*P* < 0.001) compared with normal pituitary (Fig. 3D, E, F, G, H, I, J, K, L). Furthermore, there was a strong positive correlation between CD68+ macrophage infiltration and tumoural STING expression (*P* < 0.0001) (Fig. 3M and N).

**Figure 3:**
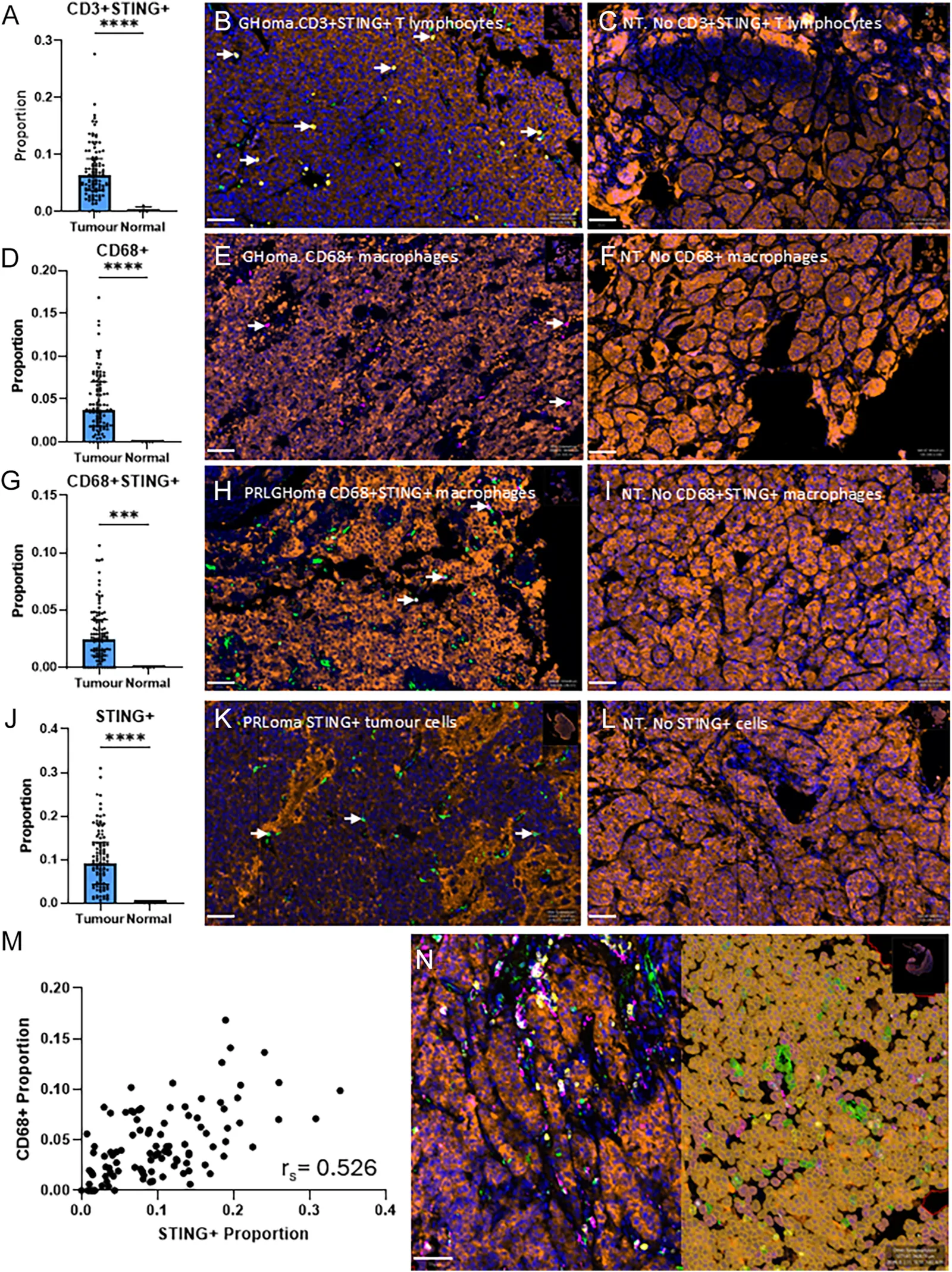
(A) Scatter plot of CD3+ STING+ T-lymphocyte proportion in tumour (*n* = 102) versus normal pituitary (*n* = 4), *P* < 0.001. (B) MIF image of somatotroph tumour representative of median tumour CD3+ STING+ T-lymphocyte positivity. Examples of CD3+ STING+ T lymphocytes indicated by white arrows. (C) MIF image of normal pituitary tissue representative of median CD3+ STING+ T-lymphocyte positivity. No CD3+ STING+ T lymphocytes are present. (D) Scatter plot of CD68+ macrophage proportion in tumour (*n* = 102) versus normal pituitary (*n* = 4), *P* < 0.001. (E) MIF image of somatotroph tumour representative of median tumour CD68+ macrophage positivity. Examples of pink CD68-positive macrophages are indicated by white arrows. (F) MIF image of normal pituitary tissue representative of median CD68+ macrophage positivity. No CD68+ macrophages are present. (G) Scatter plot of CD68+ STING+ macrophage proportion in tumour (*n* = 102) versus normal pituitary (*n* = 4), *P* < 0.001. (H) MIF image of mammosomatotroph tumour representative of median tumour CD68+ STING+ macrophage positivity. Examples of CD68+ macrophages (pink) expressing STING (green) are indicated by white arrows. (I) MIF image of normal pituitary tissue representative of median CD68+ STING+ macrophage positivity. No CD68+ STING+ macrophages are present. (J) Scatter plot of STING proportion in tumour cells (*n* = 102) versus normal pituitary (*n* = 4), *P* < 0.001. (K) MIF image of lactotroph tumour representative of median STING+ tumour cell positivity. Examples of STING+ tumour cells (green) are indicated by white arrows. (L) MIF image of normal pituitary tissue representative of median STING+ cell proportion. No STING+ pituitary cells are present. (M) Correlation plot of CD68+ macrophages and STING+ tumour cells (*n* = 102), *r*_s_ = 0.297, *P* < 0.0001. (N) MIF image of somatotroph tumour with positively correlated CD68+ and STING+ expression. Left half of image with QuPath mask off, right half of image with QuPath mask on. GHoma, somatotroph tumour; NT, normal tissue; PRLGHoma, mammosomatotroph tumour; PRLoma, lactotroph tumour. Scatter plot error bars indicate IQR. Scale bars 50 μm. All multiplex images at ×20 magnification.

### IME immune cell infiltrate and clinical phenotype

CD3+ T lymphocyte and CD68+ STING+ infiltration were significantly different for the GH excess (acromegaly), lactotroph tumour and thyrotroph tumour diagnoses, *P* = 0.041 (Fig. 4A). There were no significant differences in CD4+, CD8+, CD3+STING+ T lymphocytes, CD20+ B lymphocytes, CD68+ or tumoural STING expression between GH excess, lactotroph tumour and thyrotroph tumour (Fig. 4B, C, D, E, F, H). There were borderline significant differences in CD68+STING+ macrophage infiltrate across tumour subtypes (Fig. 4G).

**Figure 4:**
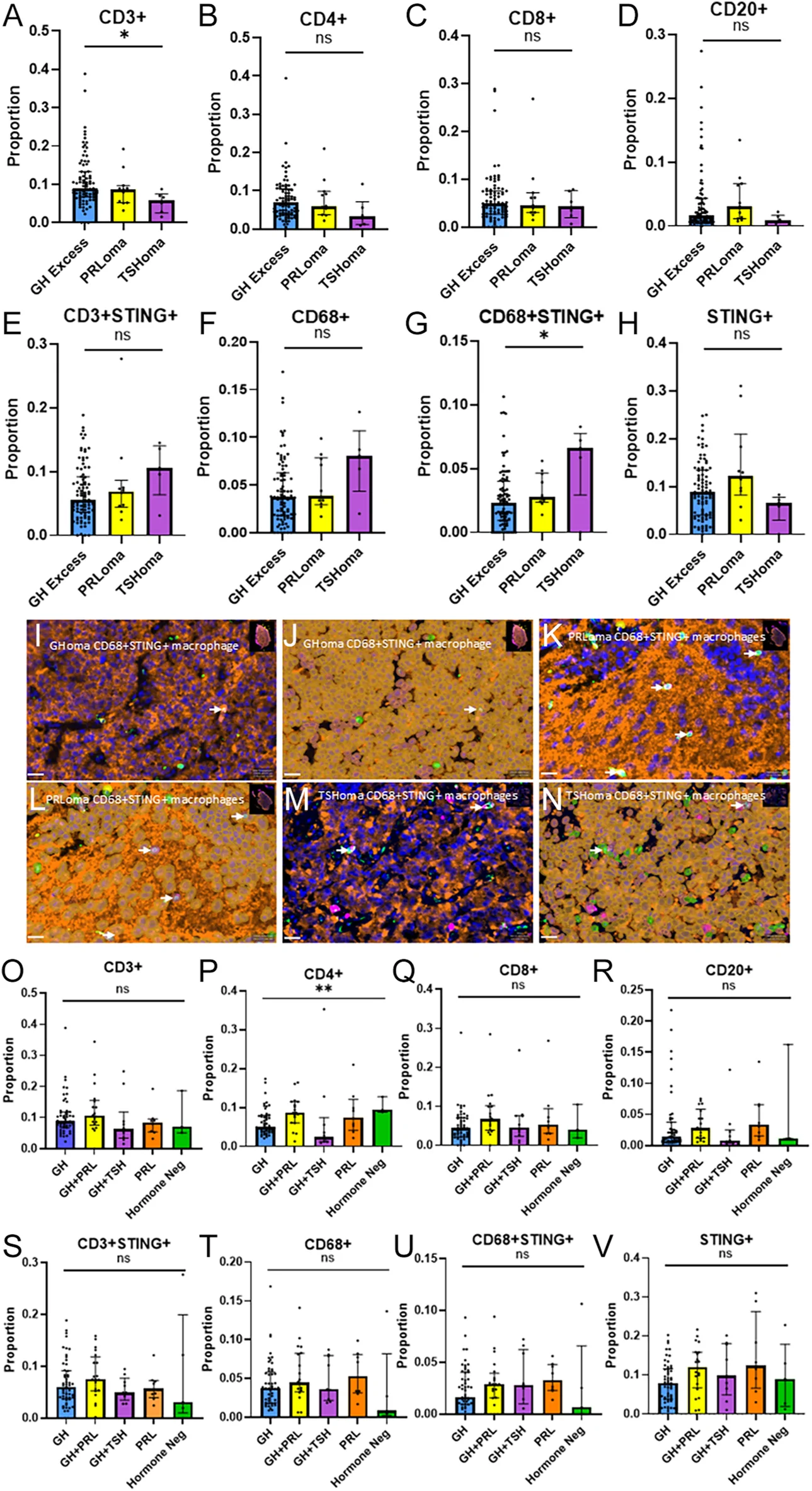
(A, B, C, D, E, F, G, H) Scatter plots of immune cell infiltrates in tumours associated with GH excess (*n* = 83), lactotroph tumour (*n* = 11), thyrotroph tumour (*n* = 6). (A) CD3+ T lymphocytes, *P* = 0.041. (B) CD4+ T, *P* = 0.041. (C) CD8+ T lymphocytes, *P* = 0.923. (D) CD20+ B lymphocytes, *P* = 0.102. (E) CD3+ STING+ T lymphocytes, *P* = 0.224. (F) CD68+, *P* = 0.107. (G) CD68+ STING+ macrophages, *P* = 0.049. (H) STING+ tumour cells, *P* = 0.081. (I) MIF image of a somatotroph tumour representative of CD68+ STING+ macrophage infiltrate without QuPath mask. CD68+ STING+ macrophages, white arrows. (J) Same somatotroph tumour as in (I) with QuPath mask on. (K) MIF image of a lactotroph tumour representative of CD68+ STING+ macrophage infiltrate without QuPath mask; CD68+ STING+ macrophages, white arrows. (L) Same lactotroph tumour as in (K) with QuPath mask on. (M) MIF image of a thyrotroph tumour representative of CD68+ STING+ macrophage infiltrate without QuPath mask; CD68+ STING+ macrophages, white arrows. (N) Same thyrotroph tumour as in (M) with QuPath mask on. CD68+ STING+ macrophages, white arrows. (O, P, Q, R, S, T, U, V) Scatter plots of immune cell infiltrates in tumours according to anterior pituitary hormone IHC. (O) CD3+ T lymphocytes, *P* = 0.280. (P) CD4+ T lymphocytes, *P* = 0.006. (Q) CD8+ T-lymphocyte infiltrates, *P* = 0.235. (R) CD20+ B lymphocytes, *P* = 0.087. (S) CD3+STING+ T lymphocytes, *P* = 0.510. (T) CD68+ macrophages, *P* = 0.172. (U) CD68+STING+ T lymphocytes, *P* = 0.355. (V) STING+ tumour cell positivity, *P* = 0.126. Error bars indicate IQR. GHoma, somatotroph tumour; PRLoma, lactotroph tumour; TSHoma, thyrotroph tumour; GH, growth hormone; PRL, prolactin; TSH, thyroid-stimulating hormone; Hormone Neg, hormone negative. One TSH+ and one ACTH+ tumour omitted from IHC statistical analysis due to small numbers. Scale bars 20 μm. Multiplex images at ×40 magnification.

### IME immune cell infiltrate and anterior pituitary hormone immunohistochemistry

When further refining diagnosis by anterior pituitary hormone IHC, there were significant differences in CD4+ T lymphocytes (*P* = 0.006), but no other significant differences in the PitNET-IME biomarkers were examined in this study (Fig. 4O, Q, R, S, T, U, V).

### IME immune cell infiltrate and tumour invasiveness

There was no significant difference in immune cell infiltrate (CD3, CD4, CD8, CD20, CD68) or in STING tumour cell expression between invasive and non-invasive tumours (Supplementary Fig. 3A, B, C, D, E, G, H).

### IME immune cell infiltrate and Ki-67 PI

There were no significant correlations between CD3+, CD3STING+, CD4+, CD8+ T lymphocytes, CD20+ B lymphocyte infiltration or STING+ tumour-cell positivity with whole-face section assessment of Ki-67 PI (Supplementary Fig. 4A, B, C, D, E, H). There was a weak positive correlation between CD68+ (*r*_s_ = 0.305, *P* = 0.003) and CD68+ STING+ macrophage (*r*_s_ = 0.263, *P* = 0.010) infiltration with Ki-67 PI (Supplementary Fig. 4F and G).

### PitNET-IME immune cell infiltrate and *AIP* mutation status

In this cohort, there were four patients with heterozygosity for the constitutional pathogenic *AIP* R304* variant from whom tumour tissues were successfully retrieved for analysis. Their tumours were compared with tumours from patients who had undergone genetic testing and were found to be negative for constitutional pathogenic *AIP* variants (*n* = 11). Results suggested that *AIP*-mutated tumours may have more CD8+ T lymphocytes, CD68+, CD68+ STING+ macrophages and more STING+ tumour cells compared with *AIP*-negative tumours (Fig. 5A, B, C, D, E, F, G, H, I).

**Figure 5:**
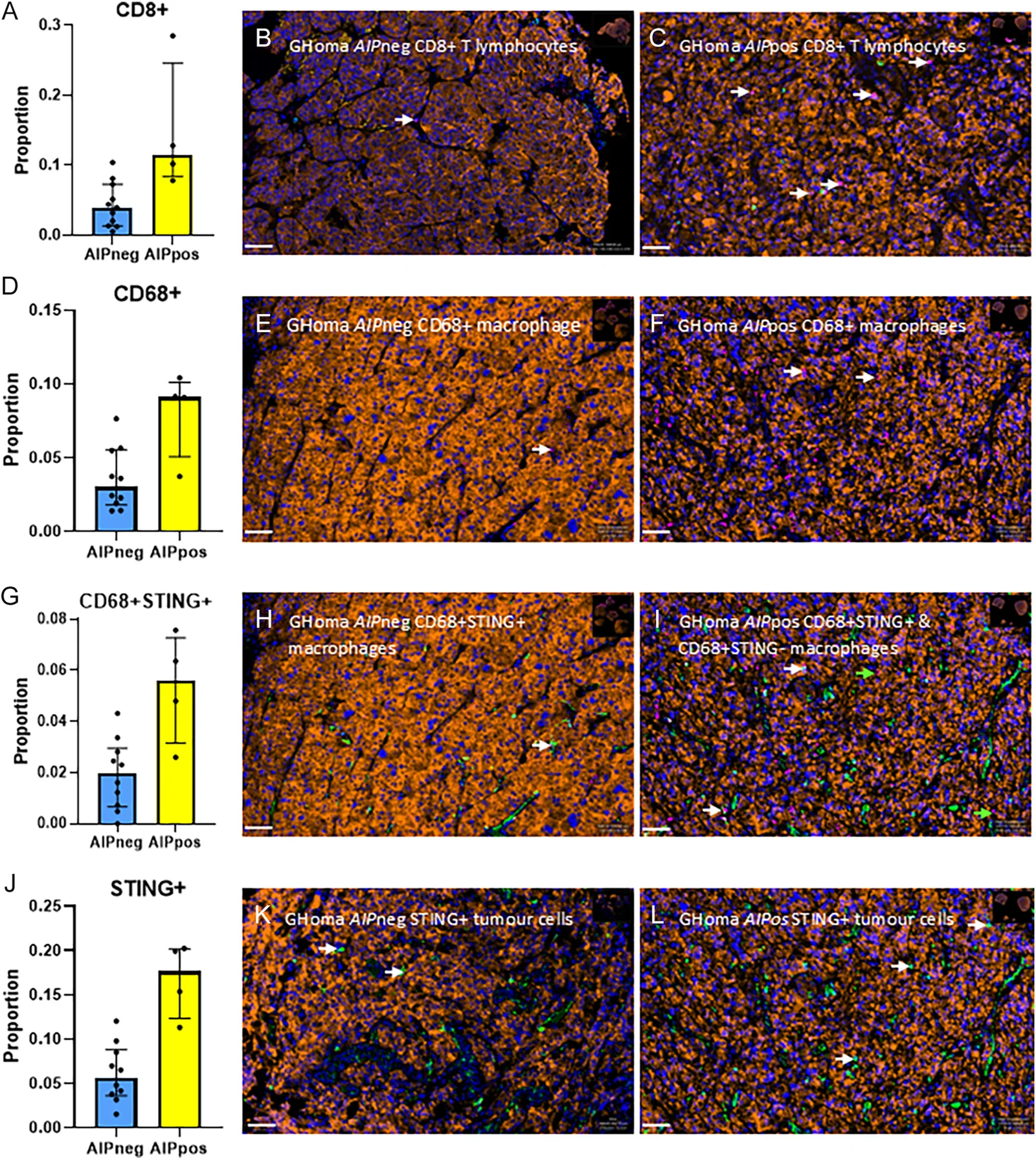
(A) Scatter plot of CD8+ T-lymphocyte proportion in AIP mutation-negative (AIPneg) somatotroph tumour (*n* = 11) versus AIP mutation-positive (AIPpos) somatotroph tumour (*n* = 4). (B) MIF image of somatotroph tumour representative of median AIPneg tumour CD8+ T-lymphocyte positivity. Example of pink CD8+ T lymphocyte indicated by white arrows. (C) MIF image of somatotroph tumour representative of median AIPpos tumour CD8+ T-lymphocyte positivity. Examples of multiple pink CD8+ T lymphocytes are indicated by white arrows. (D) Scatter plot of CD68+ macrophage proportion in AIP mutation-negative (AIPneg) somatotroph tumour versus AIP mutation-positive (AIPpos) somatotroph tumour. AIPneg *n* = 11, AIPpos *n* = 4. (E) MIF image of somatotroph tumour representative of median AIPneg tumour CD68+ macrophage positivity. Examples of pink CD68-positive macrophage indicated by white arrows. (F) MIF image of AIPpos tumour representative of median CD68+ macrophage positivity. Examples of pink CD68-positive macrophage indicated by white arrows. (G) Scatter plot of CD68+ STING+ macrophage proportion in AIPneg somatotroph tumour versus AIPpos somatotroph tumour. AIPneg *n* = 11, AIPpos *n* = 4. (H) MIF image of the same AIPneg somatotroph tumour shown in (E) with STING channel switched on. Example of CD68+ macrophages (pink) expressing STING (green) indicated by white arrows. (I) MIF image of the same AIPpos somatotroph tumour shown in (F) with STING channel switched on. Examples of CD68+ STING+ macrophages indicated by white arrows. Examples of CD68+ STING- macrophages indicated by green arrows. (J) Scatter plot of STING proportion in AIP mutation-negative (AIPneg) somatotroph tumour versus AIP mutation-positive (AIPpos) somatotroph tumour. AIPneg *n* = 11, AIPpos *n* = 4. (K) MIF image of somatotroph tumour representative of median AIPneg tumour STING+ positivity. Examples of STING+ tumour cells are indicated by white arrows. (L) MIF image of AIPpos tumour representative of median tumour STING+ positivity. Examples of STING+ tumour cells indicated by white arrows. Error bars indicate IQR. *P* values not calculated due to small numbers. Scale bars 50 μm. All multiplex images at ×20 magnification.

### PiTNET-IME immune cell infiltrate and effect of somatostatin receptor ligand (SRL) therapy

In this study, a subgroup of patients with acromegaly received treatment with somatostatin receptor ligand preoperatively, thus facilitating some insight into the potential effect of SRL on the PitNET-IME. There were 13 patients treated with SRL preoperatively with tumour available for MP1 analysis and 16 tumours treated with SRL preoperatively with tumour available for MP2 analysis. Their tumours were compared with those who did not receive preoperative SRL therapy (MP1 *n* = 65, MP2 *n* = 84). There was no significant difference in CD3+ (*P* = 0.596), CD4+ (*P* = 0.624), CD8+ (*P* = 0.471) T-lymphocyte infiltration or CD20+ B lymphocyte infiltration (*P* = 0.616) in tumours treated with SRL preoperatively compared with those that were not (Supplementary Fig. 2A, B, C, D). Similarly, when examining MP2, CD68+ (*P* = 0.930), CD68+ STING+ (*P* = 0.386) macrophage infiltration and STING+ tumour-cell positivity (*P* = 0.099) were not significantly higher in tumours treated with SRL preoperatively compared with those that were not (Supplementary Fig. 2E, F, G, H).

## Discussion

This study demonstrates for the first time the application of multiplex immunofluorescence and artificial intelligence in PitNETs for the simultaneous digital quantification of multiple biomarkers and supports recently published data suggesting innate immune STING may be a new biomarker of interest. The workflow was challenging, with samples requiring the implementation of meticulous quality control measures. Both MP1 and MP2 had the CD3 T-lymphocyte marker, and although there was no significant difference in the CD3 expression between matched samples (Fig. 2C), there was a weak positive correlation between them (Fig. 2D). In MP1, CD3 was located in position one paired to Opal 480 and in MP2 in position two paired to Opal 520 (Fig. 1A and B). Overcoming excessive autofluorescence from Opal 520 was particularly challenging. Differences in position within panels and autofluorescence may account for the weak (rather than strong/very strong) positive correlation between panels and reflect one of the many challenges and considerations in MP design.

This study indicates that PitNETs have a higher number of total macrophages, more STING-positive macrophages, more STING-positive T lymphocytes and more tumoural STING expression compared with normal pituitary. Tumoural STING expression also had a strong positive correlation with CD68+ macrophage infiltration. STING activation with subsequent macrophage recruitment to the PitNET-IME could, therefore, be a process warranting further investigation. The cGAS–STING pathway has been investigated in other neuroendocrine neoplasia, namely Merkel cell carcinoma and small cell lung carcinoma (29, 30). Both of these neuroendocrine carcinomas are characterised by low or absent STING expression and have a markedly different phenotype from PitNETs (29, 30). In a mouse model, intratumoural administration of cGAMP resulted in cGAS–STING pathway activation and associated M1 macrophage polarisation (31). A similar mechanism of increased STING activation and antitumoural CD68 macrophage recruitment may contribute to the indolent phenotype observed in PitNETs. Similarly, the cGAS–STING pathway signals interleukin-6-induced cellular senescence in a rat somatotrophinoma pituitary cell line, and this may also contribute to the indolent nature of PitNETs (32). Further work on macrophage subtyping and mechanistic studies may help unravel this complex relationship.

There were significant differences in CD3+ T lymphocyte and CD68+ STING+ macrophages between PitNET subtypes when considered according to functioning status (Fig. 4A and G); however, *P* values were borderline (0.041 and 0.049, respectively), and this difference was not observed when stratifying the cohort according to anterior pituitary hormone expression. Consequently, conclusions should be guarded; STING expression in CD3+ T lymphocytes and CD68+ macrophages could be ubiquitous across the PitNET subtypes examined in this study.

Tumour invasiveness can be a feature of aggressive PitNETs, and there were no significant differences in immune cell infiltrate or STING expression between invasive and non-invasive PitNETs confined to the sella and suprasellar space in the present study (Supplementary data). Similar observations have previously been made in somatotroph tumours, but in NF-PitNETs the pattern seems to be different (8, 9, 33, 34). The majority of NF-PitNETs are gonadotroph tumours, and this implies that invasion in this tumour subtype may be more heavily mediated by PitNET-IME composition.

The Ki-67 PI is a longstanding biomarker in PitNETs, and it is particularly relevant for aggressive tumours (35, 36). In the current cohort, there were no strong correlations found between the PitNET-IME immune biomarkers and Ki-67 expression (Supplementary data). Previous data revealed that leucocytes were more prevalent in PitNETs with Ki-67 PI >3%; however, there was no information on how Ki-67 PI was assessed, and the significance did not persist when considering CD3+, CD4+ and CD8+ T-lymphocyte subtypes (37). Combined, the data indicate that immune cell infiltrate does not have a strong relationship with proliferative activity in the indolent PitNET cohorts reported to date.

Similarly, the PitNET-IME of SRL-treated tumours does not appear to be significantly different to SRL-naïve tumours, although an acknowledged limitation is that these were unpaired comparisons. Associations between SRL treatment and the PitNET microenvironment have previously been reported. These studies asked different questions to the current study but showed that macrophage infiltration did not differ between SRL-resistant and SRL-responsive tumours and CD8+ T lymphocytes were more prevalent in SRL-responsive tumours (9, 11). Conclusions should be cautious, but the findings suggest that SRLs do not appear to alter tumoural infiltrating lymphocytes or macrophages.

*AIP*-mutated PitNETs are rare and are recognised to be more clinically problematic than their sporadic counterparts (38). The seemingly higher levels of CD8+ T lymphocytes and CD68+ macrophages in *AIP*-mutated PitNETs are consistent with previous work which employed manual brightfield assessment, although numbers in the current study are very small (7). In this current study examining human tissue, *AIP*-mutated PitNETs appeared to have higher tumoural STING expression. Preliminary data from *Aip*^−/−^ mouse embryonic fibroblasts showed the reverse (39). Although the data appear conflicting, it has been established that the cGAS–STING pathway may behave in an organ- and species-specific manner (40, 41). The seemingly higher STING expression coupled with higher CD8 T lymphocytes and CD68 macrophages may suggest that there is additional DNA damage, cellular senescence and autophagy in *AIP*-mutated PitNETs. This will require validation in larger cohorts which will be challenging.

The pituitary database in the current study is a retrospectively generated neurosurgical dataset; therefore, some of the data are incomplete or unavailable, for example imaging characteristics and transcription factor expression to further refine histological diagnosis. In this exploratory analysis, there were multiple comparisons performed with a potential for false discovery. This could be considered a criticism; however, without such comparisons, there is the potential to miss true-positive findings in biomarker assessment (42). Reassuringly, the majority of tests regarding CD3, CD4, CD8 and CD20 biomarkers were non-significant, while CD68 and STING repeatedly met significance when biologically and clinically informed questions were asked.

The present study was undertaken in a laboratory by a research group with experience in multiplex immunofluorescence and digital image analysis. The time and resources required to undertake such work are costly and require significant computing power. The machines used to undertake the image analysis included an 8-core Intel Xeon W-2145 CPU at 3.7 GHz clock speed, along with 128 GB of 2,666 MHz DIMM RAM. Analysing multiplex immunofluorescence images has substantially higher computing cost than traditional haematoxylin and eosin due to the additional channels for each marker (43). The significant resources required to analyse these PitNET subtypes make this study more difficult to reproduce, and may pose a challenge to high-throughput clinical translation, something that may be addressed through cloud-based solutions (44).

Somatotroph tumours, mammosomatotroph tumours, thyrotroph tumours and operated lactotroph tumours are rare, and this study provides insights into their immune microenvironment. Multiplex immunofluorescence is a powerful technique, and with tyramide signal amplification, it is more sensitive than the standard immunohistochemistry studies interrogating the PitNET-IME to date. Coupled with the digitisation and automation of workflow, this holds promise for future studies into such tumours. A recurrent challenge in histopathological studies of pituitary tissue is the acquisition of a normal control. Previous studies examining PitNET biomarkers have resorted to autopsy material for normal pituitary tissue; however, there is rapid degradation of protein, DNA and RNA following death (45). This has the potential to amplify any differences in biomarker expression between tissues retrieved from living compared with deceased subjects. Paracrine changes are known to occur in normal pituitary tissue adjacent to functioning tumours, for example Crooke cell hyaline change. The utilisation of tissue from live patients with Rathke’s cleft cysts is a strength in this study and avoids this potential confounder.

The work undertaken to date will support future studies into the PitNET-IME, including spatial analysis and characterisation of other PitNET subtypes using the same digital image analysis pipeline. With new European Society for Endocrinology guidance now suggesting immunotherapy as second-line therapy for patients with PitNETs progressing on temozolomide, it will be intriguing to see whether histopathological analysis of the PitNET-IME could be utilised to predict immunotherapy response or indeed identify novel therapeutic options (1).

## Conclusion

Multiplex immunofluorescence and application of deep learning algorithms to interrogate digital images of PitNETs is laborious, but feasible. This study using novel image analysis techniques reinforces existing data that indicate macrophages have important roles in PitNET pathogenesis. Innate immune STING expression is upregulated in lactotroph tumours, thyrotroph tumours and PitNETs associated with growth hormone excess compared with normal pituitary and is strongly positively correlated with CD68+ macrophage infiltration. These findings indicate that macrophages and STING could be relevant to the PitNET-IME. Future investigation of STING and associated processes such as DNA damage, cellular senescence and autophagy could be considered.

## Supplementary materials



## Declaration of interest

Jacqueline A James is an unpaid member of the Breast Cancer Now Tissue Bank Advisory Committee and an unpaid Non-Executive Director of Biobank Ireland Trust.

## Funding

This work was supported by the Health and Social Care Research and Development Division, Public Health Agency (EAT/5498/18) and the Irish Endocrine Society.

## Author contribution statement

PBL, MK and JAJ conceptualised this study. PBL, AM and AVP curated the data. PBL, AM, KDM, CG, HJ, FC, ER, MK and JAJ formally analysed and investigated the study data. PBL, SJH, MK and JAJ acquired the funding. PBL, AM, AVP, FC, ER and HJ investigated the study data. PBL, AM, AVP, KDM, CG, HJ, ER, SGC, MK and JAJ designed the methodology. PBL, AVP, CG, SGC and JAJ administered the project. PBL, CG, EGH, SJH and JAJ dealt with resources for this study. PBL, AVP and KDM worked with the software. SJH, SGC, MK and JAJ supervised this study. PBL, AM, SGC and JAJ validated the study details. PBL visualised this study and wrote the original draft of this article. PBL, AM, AVP, KDM, CG, HJ, EGH, FC, ER, SC, SJH, MK, SGC and JAJ wrote, reviewed and edited this article.

## Ethics statement

Data were retrospectively retrieved from patient records and held within an audit and quality improvement database registered with the Belfast Health and Social Care Trust (approval 6057). The linkage of de-identified clinicopathological data and samples was undertaken using established procedures between the Northern Ireland Biobank and the Northern Ireland Cancer Registry. The study was conducted according to the National Institute for Healthcare Research Good Clinical Practice guidelines and the Declaration of Helsinki and its later amendments or comparable ethical standards. All surgical samples were removed from patients with full informed consent. Use of anonymised surplus formalin-fixed paraffin-embedded samples for the multiplex immunofluorescence analysis was granted in accordance with standard operating procedures for the Northern Ireland Biobank under the study approval NIB18-0282.

## Data availability

The data that support the findings of this study are available from the corresponding author upon reasonable request.

## AI disclosure

No generative artificial intelligence (AI) tools were used in the preparation of the written or visual content of this manuscript.
