# Supplementary material for: STING expression in pituitary neuroendocrine tumour immune microenvironments
Source: Endocr Relat Cancer. 2026 Sep 28;33(9):e260385. doi: 10.1530/ERC-26-0385 (PMC13641126; doi:10.1530/ERC-26-0385)
Supplement: Supplementary file 1 [file ERC-26-0385_supplementary_figures.pdf]

Supplementary Materials

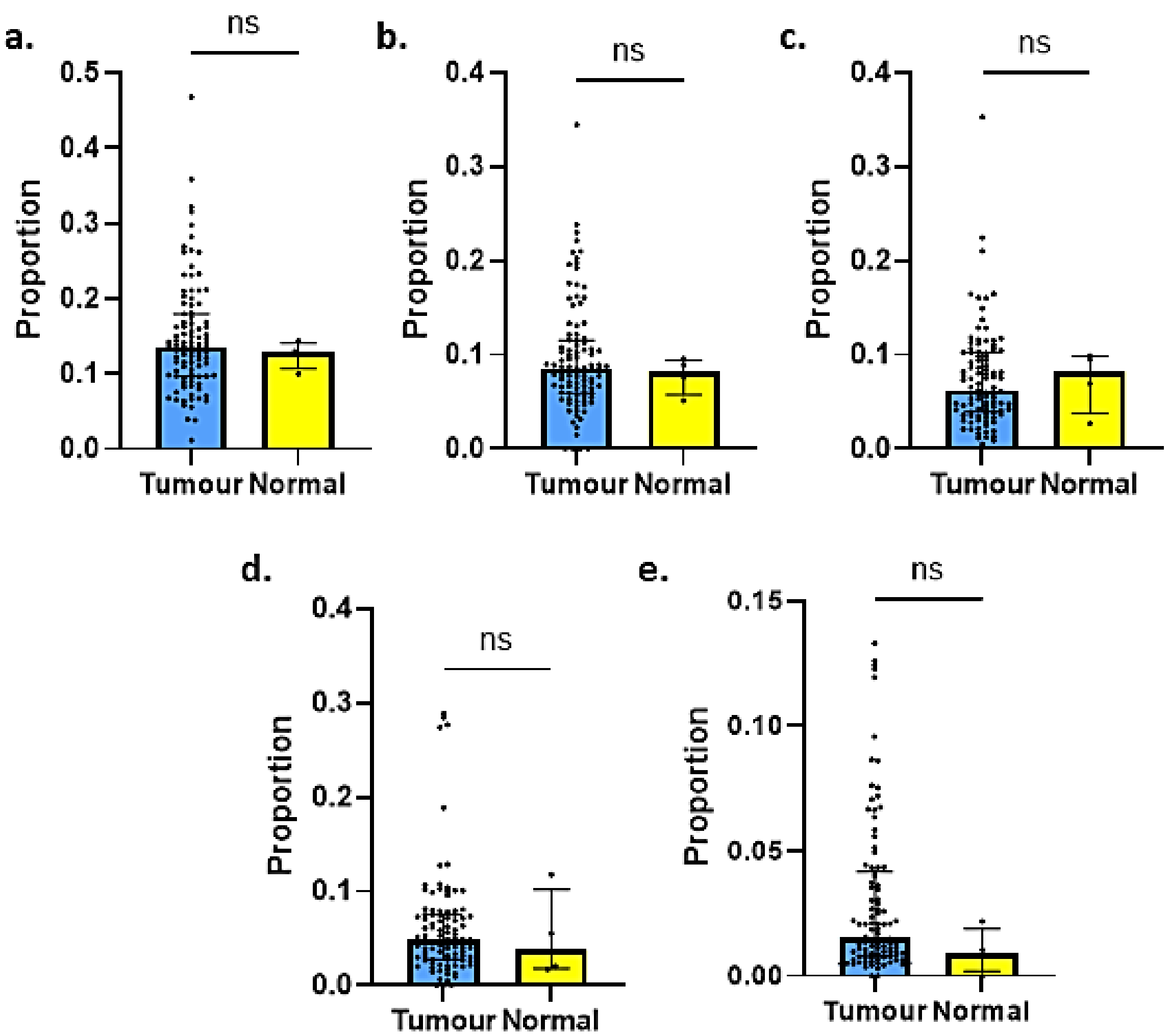

Supplementary Fig. 1 a. Total immune cell infiltrate (CD3, CD4, CD8, CD20) in tumours (*n*=99) compared to normal pituitary (*n*=4), *p*=0.687. **b.** CD3+ T lymphocytes in tumours (*n*=99) compared to normal pituitary (*n*=4), *p*=0.723 **c.** CD4+ T lymphocytes in tumours (*n*=99) compared to normal pituitary (*n*=4), *p*=0.876 **d.** CD8+ T lymphocytes in tumours (*n*=99) compared to normal pituitary (*n*=4), *p*=697 **e.** CD20+ B lymphocytes in tumours (*n*=99) compared to normal pituitary (*n*=4), *p*=0.176. Error bars indicate IQR.

IME Immune Cell Infiltrate and Clinical Phenotype

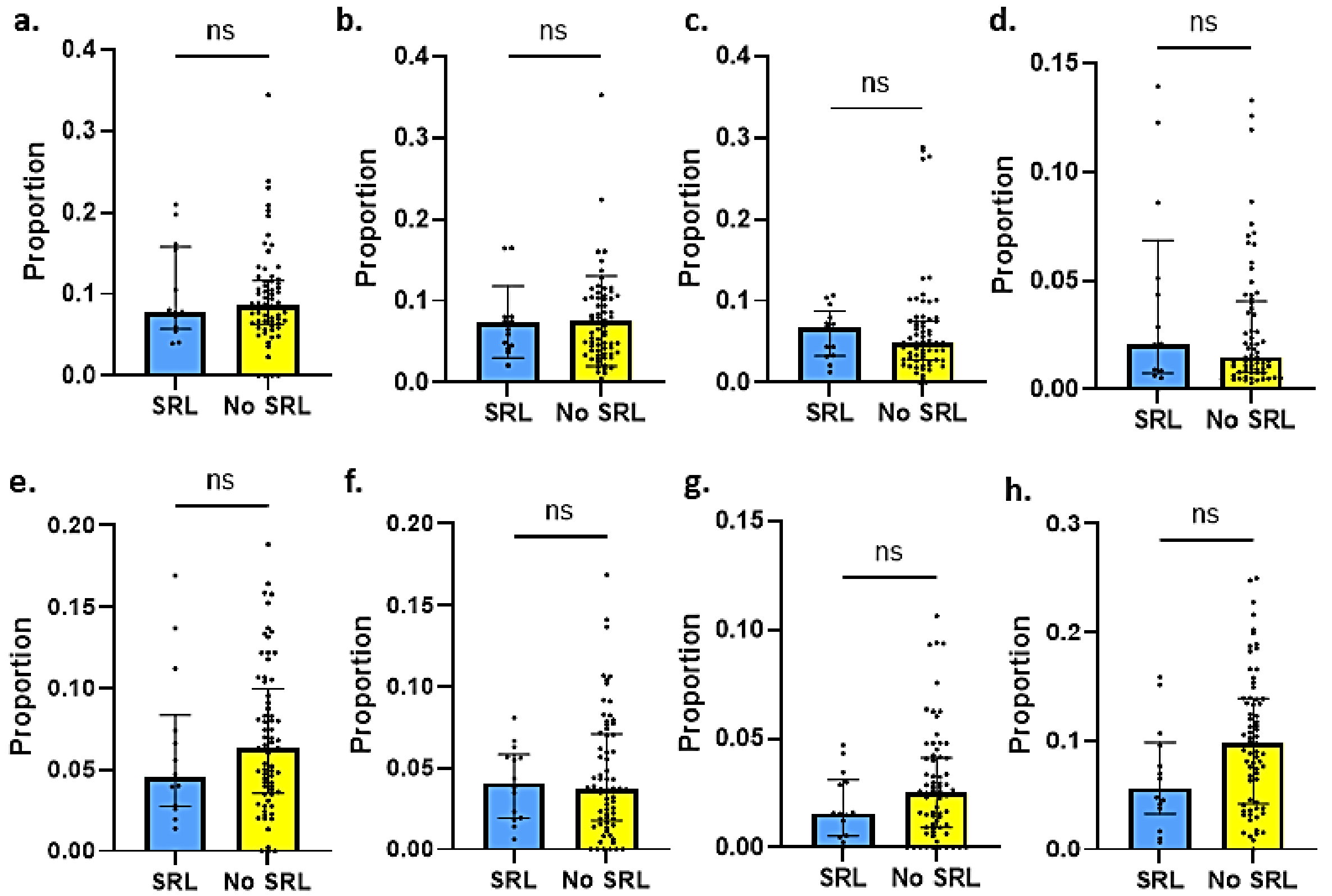

Supplementary Fig. 2 a. CD3+ T lymphocytes in tumours associated with GH excess pretreated with SRL (*n*=13) compared to those not treated with SRL (*n*=66), *p*=0.972 **b.** CD4+ T lymphocytes in tumours associated with GH excess pretreated with SRL (*n*=13) compared to those not treated with SRL (*n*=66), *p*=0.943 **c.** CD8+ T lymphocytes in tumours associated with GH excess pretreated with SRL (*n*=13) compared to those not treated with SRL (*n*=66), *p*=0.524 **d.** CD20+ B lymphocytes in tumours associated with GH excess pretreated with SRL (*n*=13) compared to those not treated with SRL (*n*=66), *p*=0.485 **e.** CD3+STING+ T lymphocytes in tumours associated with GH excess pretreated with SRL (*n*=14) compared to those not treated with SRL (*n*=69), *p*=0.444 **f.** CD68+ macrophages in tumours associated with GH excess pretreated with SRL (*n*=14) compared to those not treated with SRL (*n*=69), *p*=0.983 **g.** CD68+STING+ macrophages in tumours associated with GH excess pretreated with SRL (*n*=14) compared to those not treated with SRL

(*n*=69), *p*=0.343 **h.** STING+ tumour cells in tumours associated with GH excess treated with GH pretreated with SRL (*n*=14) compared to those not treated with SRL (*n*=69), *p*=0.069. Error bars indicate IQR

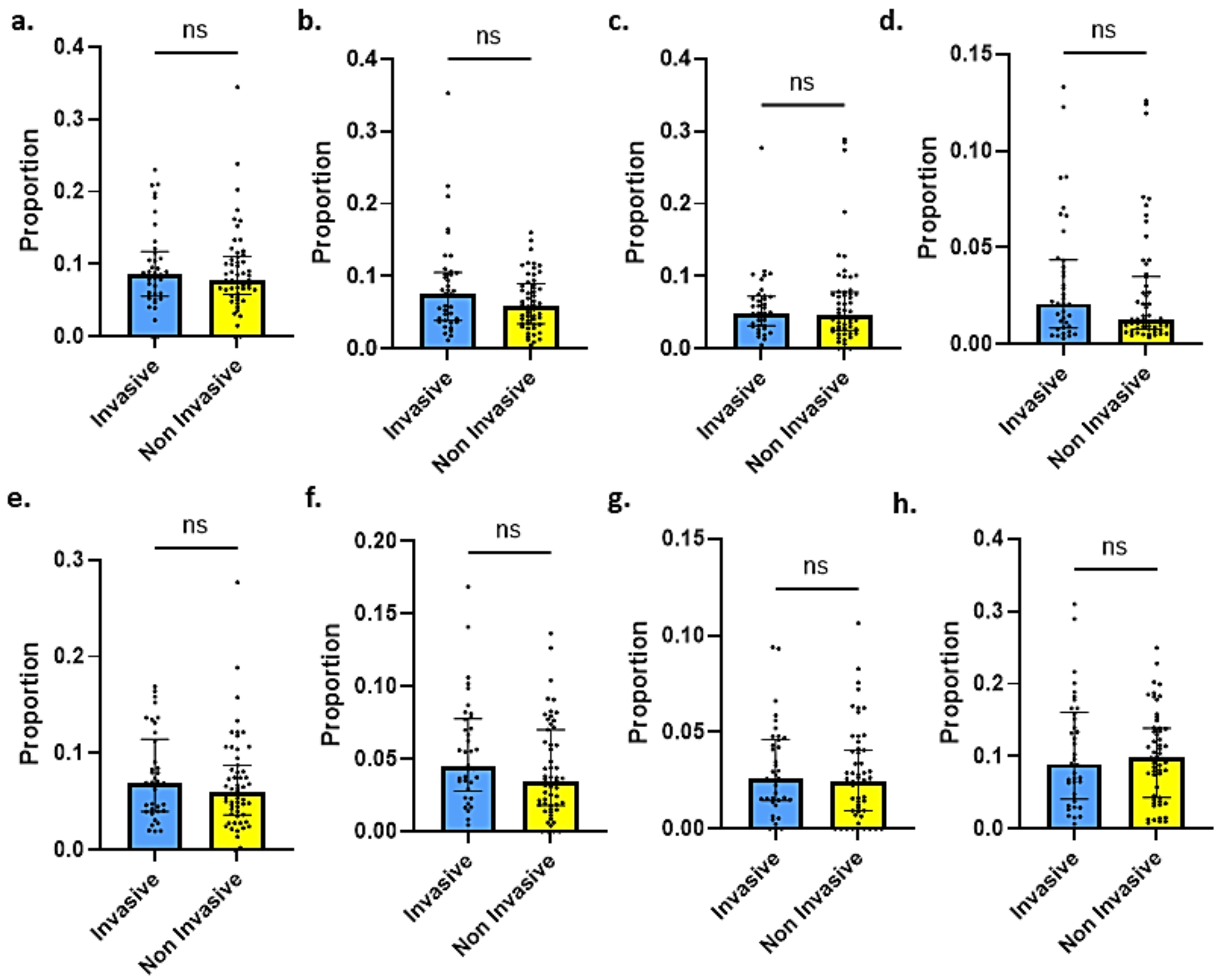

Supplementary Fig. 2 a. CD3+ T lymphocytes in invasive tumours (*n*=38), *versus* non-invasive tumours (*n*=54), *p*=0.701 **b.** CD4+ T lymphocytes in in invasive tumours (*n*=38), *versus* non-invasive tumours (*n*=54), *p*=0.207 **c.** CD8+ T in invasive tumours (*n*=38), *versus* non-invasive tumours (*n*=54), *p*=0.861 **d.** CD20+ B lymphocytes in invasive tumours (*n*=38), *versus* non-invasive tumours (*n*=54) *p*=0.322 **e.** CD3+STING+ T lymphocytes in invasive tumours (*n*=38), *versus* non-invasive tumours (*n*=55), *p*=0.487 **f.** CD68+ in invasive tumours (*n*=38), *versus* non-invasive tumours (*n*=55), *p*=0.094 **g.** CD68+STING+ macrophages in invasive tumours (*n*=38), *versus* non-invasive tumours (*n*=55), *p*=0.547 **h.** STING+ tumour cells in invasive tumours (*n*=38), *versus* non-invasive tumours (*n*=55), *p*=0.972. Error bars indicate IQR

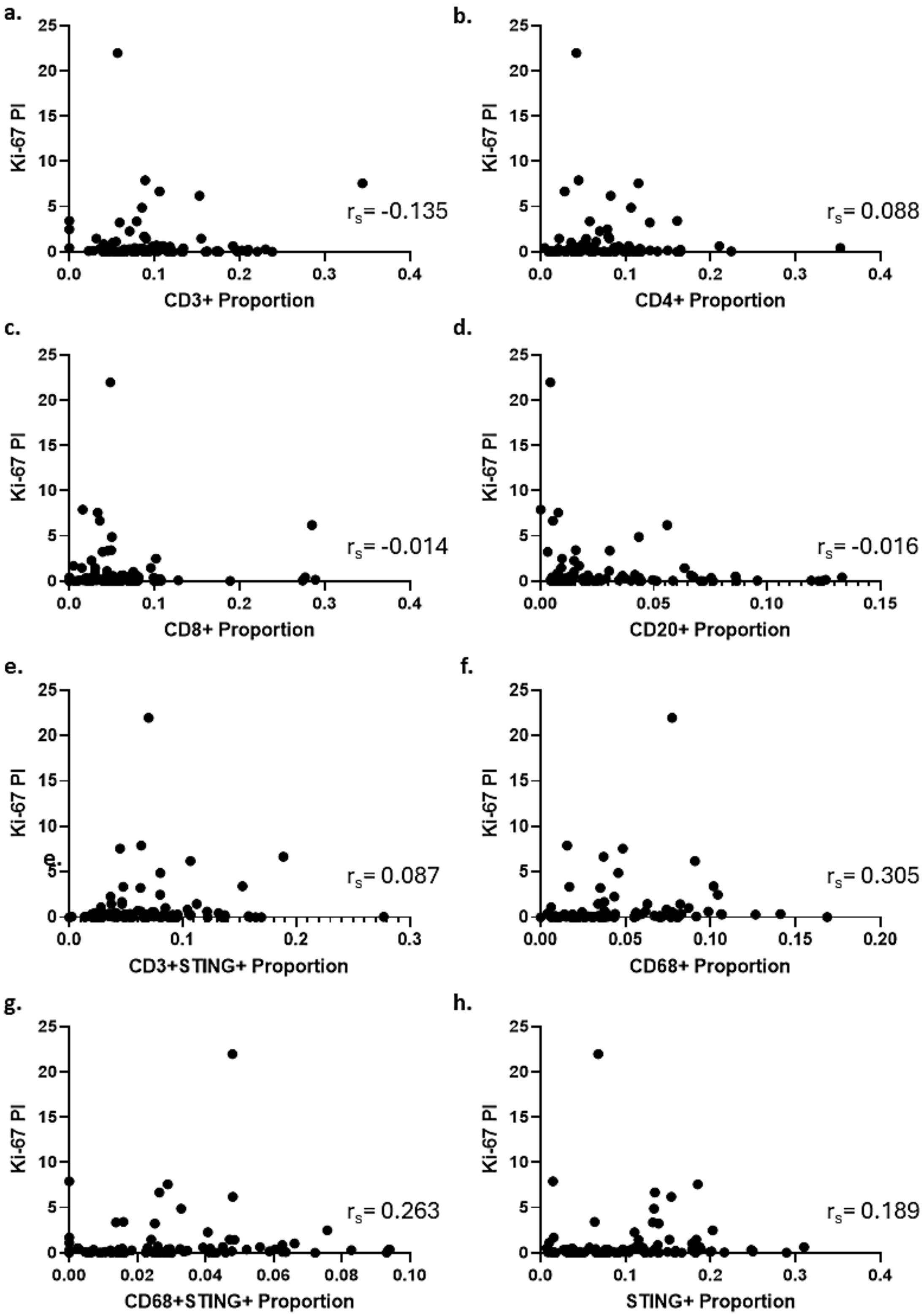

Supplementary Fig 4a. Correlation plot of Ki-67 PI and CD3+ T lymphocyte infiltration, (*n*=99), r_s=-0.135, *p*=0.191 **b.** Correlation plot of Ki-67 PI and CD4+ T lymphocyte infiltration, (*n*=99), r_s=0.088, *p*=0.396 **c.** Correlation plot of Ki-67 PI and CD8+ T

lymphocyte infiltration, (*n*=99), r_s=-0.014, *p*=0.893 **d.** Correlation plot of Ki-67 PI and CD20+ B lymphocyte infiltration, (*n*=99), r_s=-0.016, *p*=0.879 **e.** Correlation plot of Ki-67 PI and CD3+STING+ T lymphocyte infiltration, (*n*=99), r_s=0.087, *p*=0.402 **f.** Correlation plot of Ki-67 PI and CD68+ macrophage infiltration, (*n*=102), r_s=0.305, *p*=0.402 **g.** Correlation plot of Ki-67 PI and CD68+STING+ macrophage infiltration, (*n*=102), r_s=0.263, *p*=0.010 **h.** Correlation plot of Ki-67 PI and STING+ tumour cells, (*n*=102), r_s=0.189, *p*=0.067

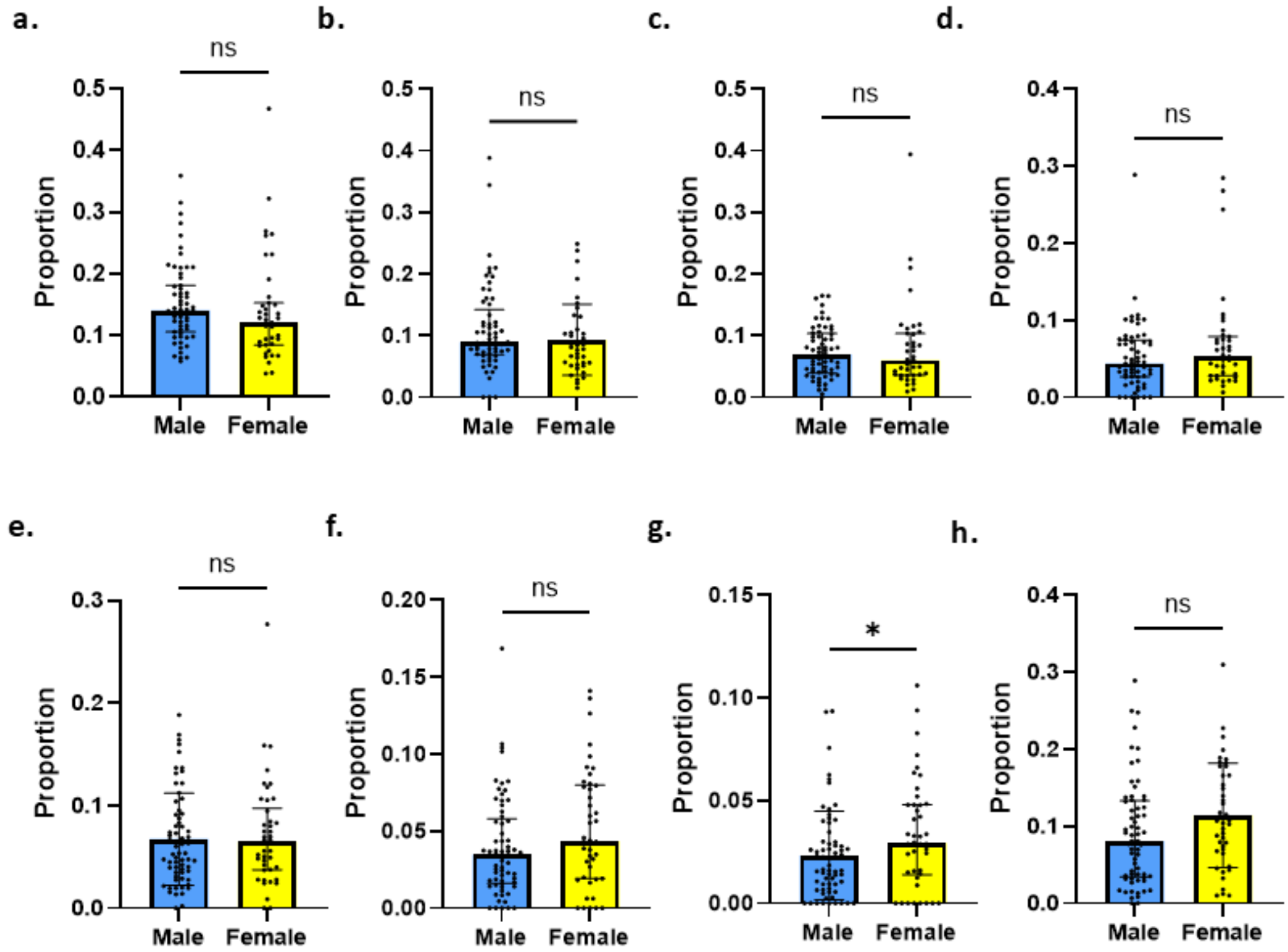

Supplementary Fig. 5a. CD3+ T lymphocytes in tumours from males (*n*=60) compared to females (*n*=39), *p*=0.723 **b.** CD4+ T lymphocytes in tumours from males (*n*=60) compared to females (*n*=39), *p*=0.723 **c.** CD8+ T lymphocytes in tumours from males (*n*=60) compared to females (*n*=39), *p*=0.723 **d.** CD20+ B lymphocytes in tumours from males (*n*=60) compared to females (*n*=39), *p*=0.723. **e.** CD3+STING+ lymphocytes in tumours from males (*n*=62) compared to females (*n*=40) **f.** CD68+ macrophages in tumours from males (*n*=62) compared to females (*n*=40) **g.** CD68+STING+ macrophages in tumours from males (*n*=62) compared to females (*n*=40) **h.** STING+ tumour cells in tumours from males (*n*=62) compared to females (*n*=40). Error bars indicate IQR.

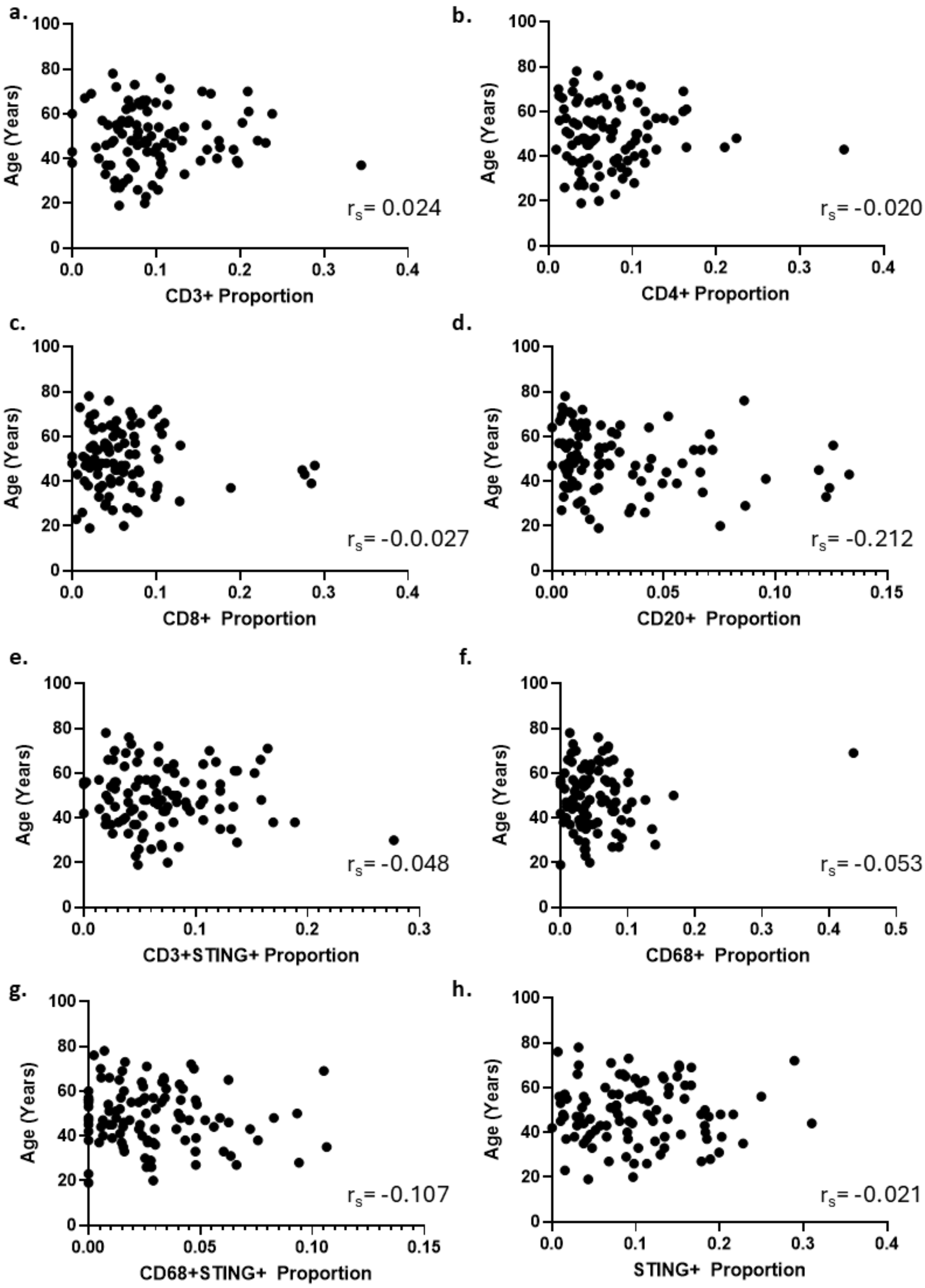

Supplementary Fig 6a. Correlation plot of age at surgery and CD3+ T lymphocyte infiltration, (*n*=99), r_s=0.024, *p*=0.811 **b.** Correlation plot of age at surgery and CD4+ T lymphocyte infiltration, (*n*=99), r_s=0.020, *p*=0.847 **c.** Correlation plot of age at surgery and CD8+ T lymphocyte infiltration, (*n*=99), r_s=-0.014, *p*=0.893 **d.** Correlation plot of age at

surgery and CD20+ B lymphocyte infiltration, (n=99), r_s=-0.212, p=0.036 **e.** Correlation plot of age at surgery and CD3+STING+ T lymphocyte infiltration, (n=99), r_s=0.048, p=0.629 **f.** Correlation plot of age at surgery and CD68+ macrophage infiltration, (n=102), r_s=-0.053, p=0.596 **g.** Correlation plot of age at surgery and CD68+STING+ macrophage infiltration, (n=102), r_s=-0.107, p=0.286 **h.** Correlation plot of age at surgery and STING+ tumour cells, (n=102), r_s=0.-0.021, p=0.834
